# Supplementary material for: Active head rolls enhance sonar-based auditory localization performance
Source: PLoS Comput Biol. 2021 May 10;17(5):e1008973. doi: 10.1371/journal.pcbi.1008973 (PMC8136848; doi:10.1371/journal.pcbi.1008973)
Supplement: S1 Text — (PDF) [file pcbi.1008973.s001.pdf]

S1 Text

In this section, we present the supporting information that are auxiliary to the manuscript. There are two section under supporting information. Section S1.1 details the 3D head pose estimation approach and the section S1.2 details the basis vector property estimation approach.

S1.1 3D head pose estimation approach

- The rotational matrix $\mathrm{R} \in \mathbb{R}^{3\times 3}$ is the composition of the unit vectors $\mathrm{V_x}$,$\mathrm{V_y}$ and $\mathrm{V_z}$ (see S1 Fig.).

$$\mathrm{R} = [\mathrm{V_x V_y V_z}] \tag{1}$$

- According to the Fick gimbal system, the azimuth, elevation and roll angles θ,ϕ and γ(in that order) can also be used to compute the rotation matrix R.

$$\mathrm{R} = \mathrm{R_z}(\theta)\mathrm{R_x}(\phi)\mathrm{R_y}(\gamma) \tag{2}$$

- The rotation angles are computed using the elements $\mathrm{r}_{i,j}; 1 \leq i, j \leq 3$ of the matrix R.

$$\theta = \tan^{-1}\Big(-\frac{\mathrm{r}_{1,2}}{\mathrm{r}_{2,2}}\Big), \tag{3a}$$

$$\phi = \sin^{-1}\big(\mathrm{r}_{3,2}\big), \tag{3b}$$

$$\gamma = \tan^{-1}\Big(-\frac{\mathrm{r}_{3,1}}{\mathrm{r}_{3,3}}\Big), \tag{3c}$$

S1.2 Basis vector property estimation

- We fit the function $\mathrm{g}(t) = \mathrm{A}\cos(2\pi \mathrm{F}(t)t + \psi) \times e^{-(\frac{t-\mathrm{t}_o}{\sigma_\mathrm{t}})^2}$ separately to the left and right components of the basis vector (see S5 Fig.). The start and end frequencies of the FM sweep is shared across the two components.
  Let,$\mathrm{f_{start}}$ $-$ start frequency, $\mathrm{f_{end}}$ $-$ end frequency, T $-$ duration. Here, the instantaneous frequency $\mathrm{F}(t) = \frac{\mathrm{m}}{2}t + \mathrm{f_{start}}$
- Interaural Level Difference (ILD)=$20\log_{10}\left(\frac{\mathrm{A_{left}}}{\mathrm{A_{right}}}\right)$
- Interaural Time Difference (ITD)=$\left(\frac{\psi_{\mathrm{left}} - \psi_{\mathrm{right}}}{2\pi \mathrm{f_c}}\right)$
- The center frequency ($\mathrm{f_c}$)=$\frac{\mathrm{f_{start}} + \mathrm{f_{end}}}{2}$
- The sweep rate (m)=$\frac{\mathrm{f_{end}} - \mathrm{f_{start}}}{\mathrm{T}}$
