# Supplementary figures and images for: Active head rolls enhance sonar-based auditory localization performance
Source: PLoS Comput Biol. 2021 May 10;17(5):e1008973. doi: 10.1371/journal.pcbi.1008973 (PMC8136848; doi:10.1371/journal.pcbi.1008973)

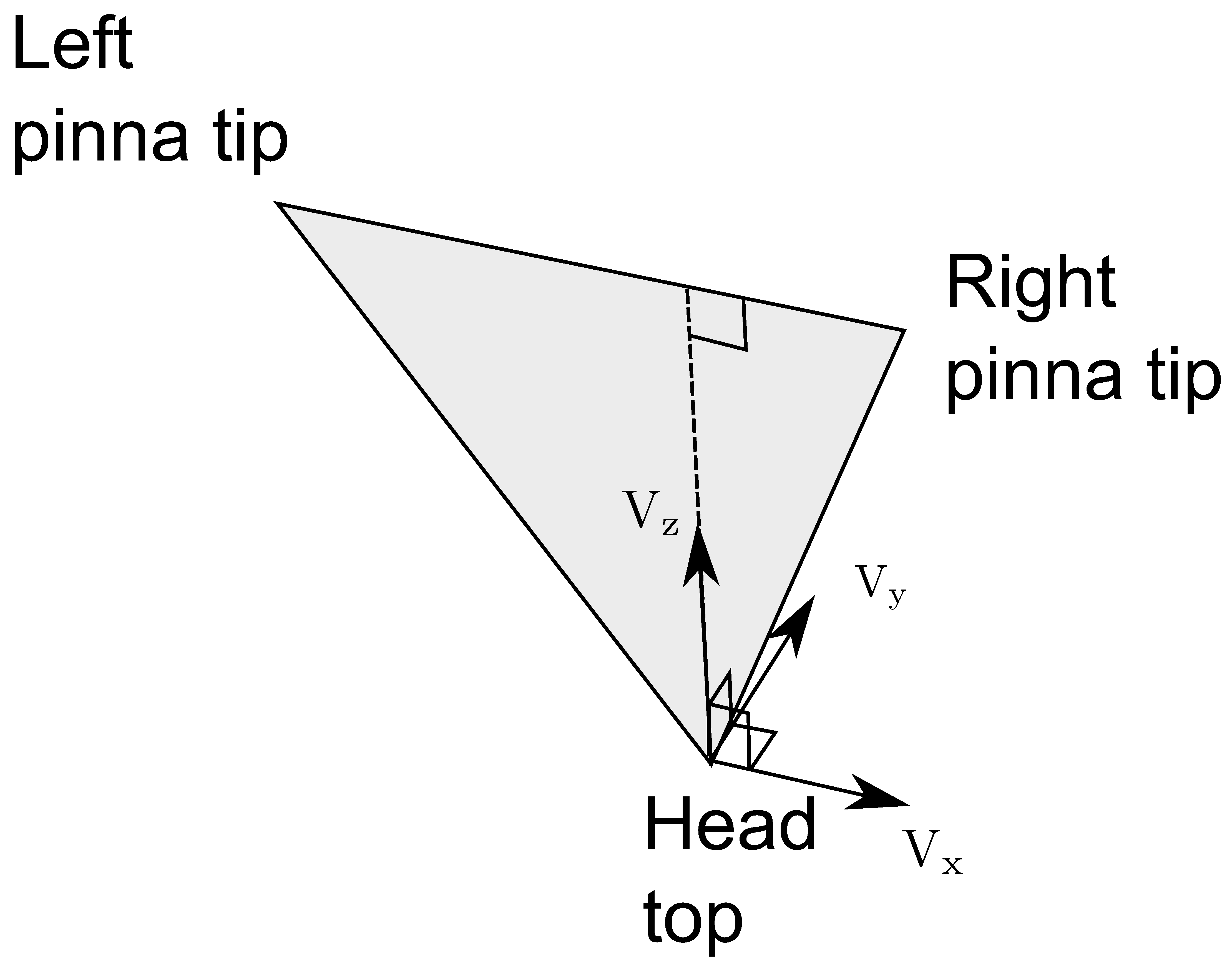

Supplement: S1 Fig — The figure shows the plane that goes through the left pinna tip, right pinna tip and the head. The vector Vz denotes the unit vector through the head and the orthocenter of the triangle. In the bat experiment, the bats adjusted inter-pinna separation. They either raise the tips of the pinnae to decrease the inter-pinna separation or lower the tips of the pinnae to increase the inter-pinna separation. We define the vector Vz perpendicular to the line through the pinna tips. Therefore, the pinna movements have a small effect on the estimation of head orientation. Also, they do not change the conclusion of our findings, namely that head rolls improve localization accuracy in the vertical plane. The vector Vy shows the unit vector perpendicular to the plane. The unit vector Vx denotes the vector perpendicular to both Vy,Vz. (TIF) [file pcbi.1008973.s002.tif]

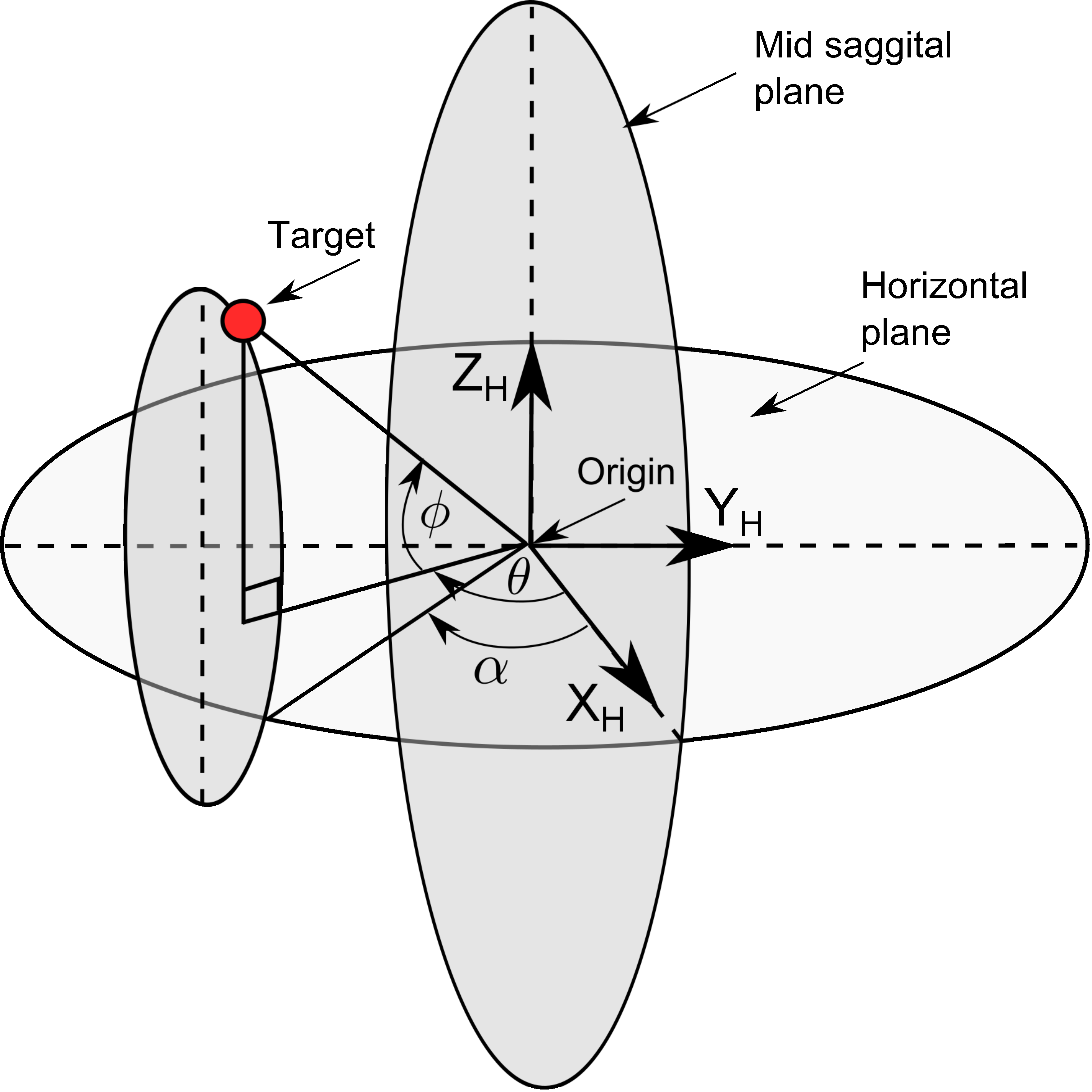

Supplement: S2 Fig — The target is located on the frontal hemisphere and 1m away from the origin. The target direction is indicated by azimuth and elevation angles θ,ϕ. The horizontal angle of the same target is indicated by α, it is defined by the intersection between the cone of confusion and the horizontal plane. The target in (θ,ϕ) in azimuth, elevation space can be transformed to (α,ϕ) in horizontal and elevation space. The axis XH indicates the forward head direction. The axis YH indicates the ear-ear direction. The axis ZH indicates the upward direction. As the bat roll the head around the head direction indicated by XH, the horizontal plane will be rotated around XH. (TIF) [file pcbi.1008973.s003.tif]

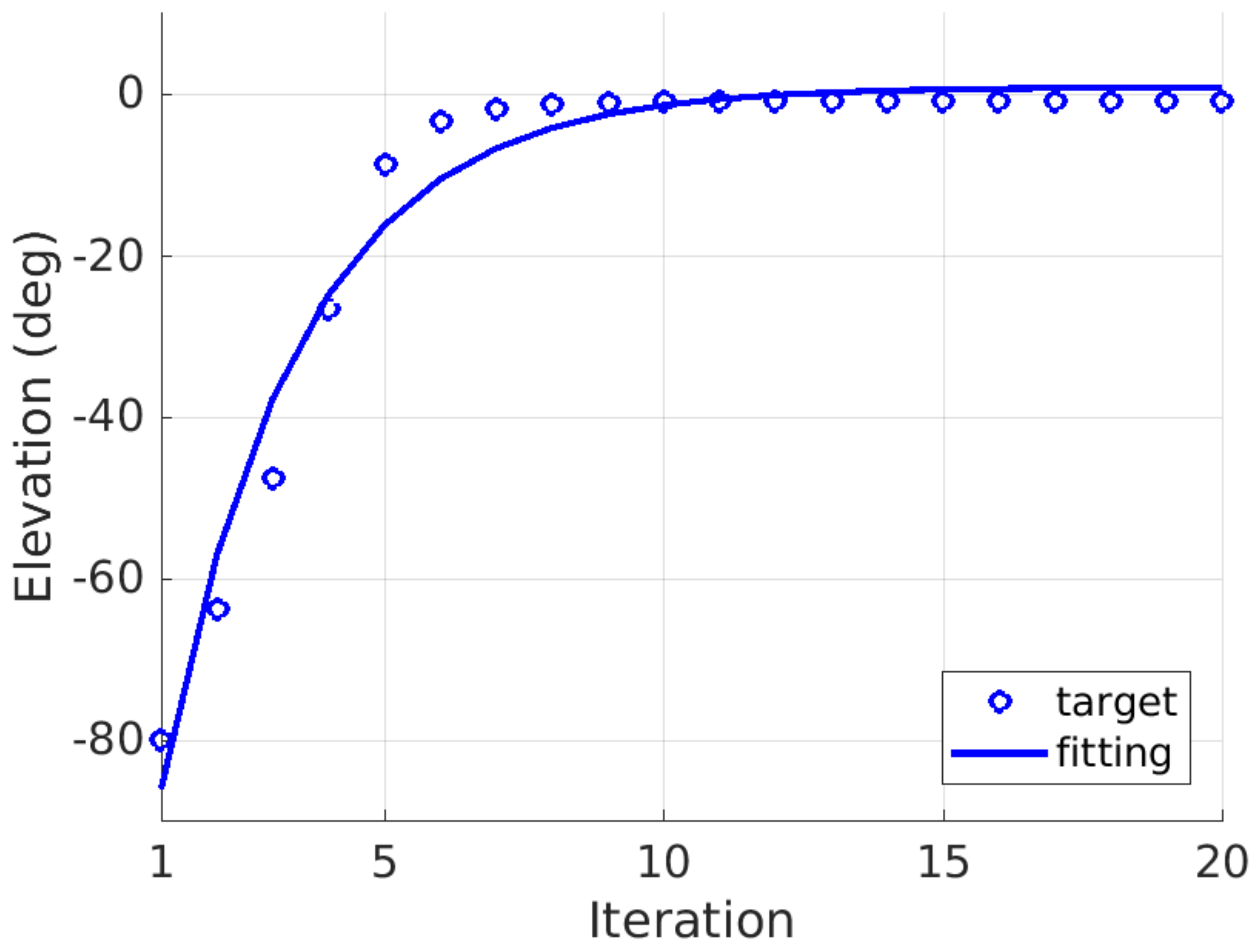

Supplement: S3 Fig — The figure shows the target elevation in the head-centric coordinates (blue dot), with the least squares fitting (blue line) of the step response s(t) = Ae−t/τ + B. For the illustrated example, A = -89.90 deg, B = 0.74 deg and τ = 2.46 iteration. (TIF) [file pcbi.1008973.s004.tif]

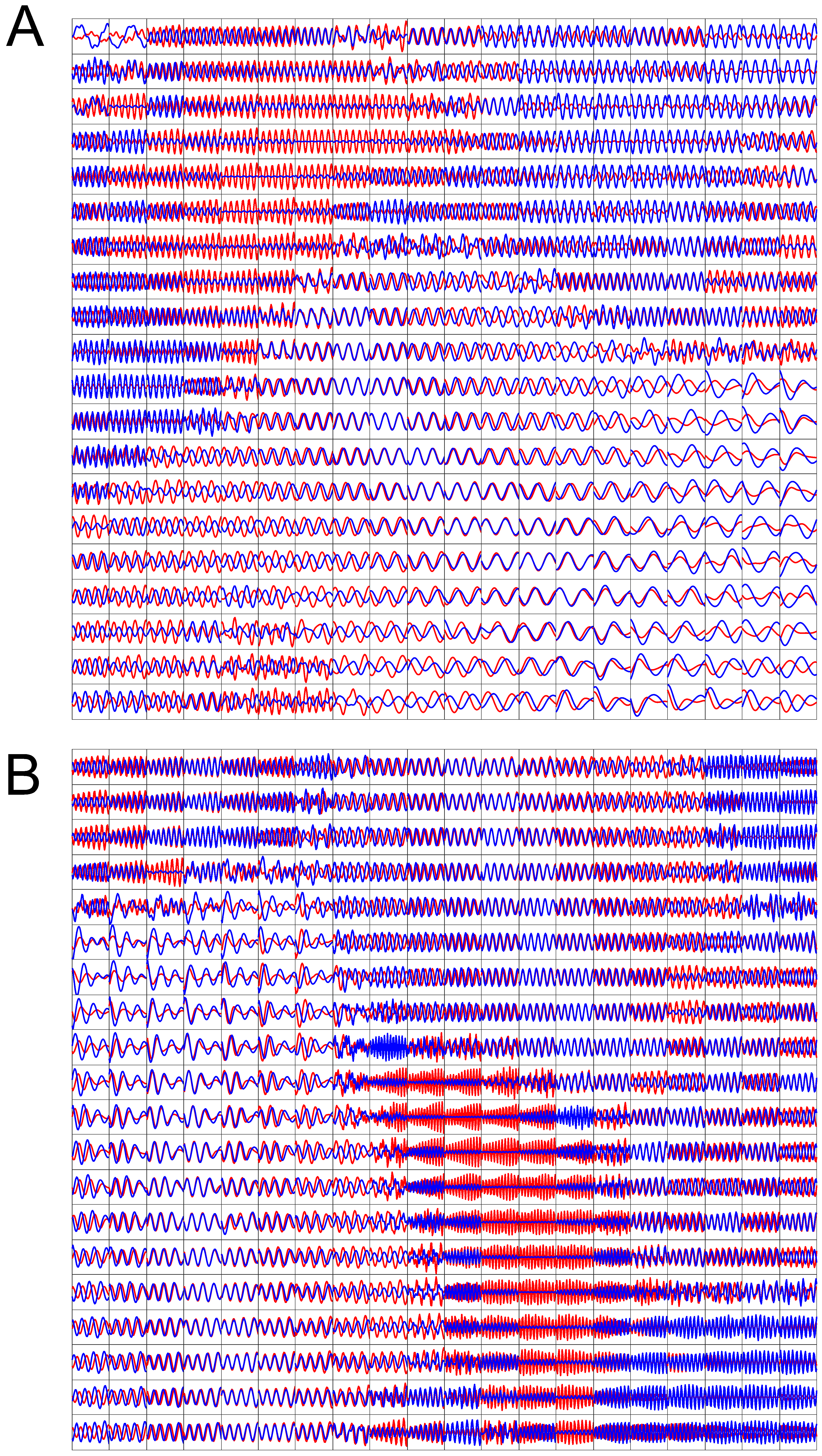

Supplement: S4 Fig — (A,B) The figures show the learned dictionary of 400 fine and coarse basis vectors. The basis vectors are shown in a 20 × 20 grid. Each element in the grid shows the left and right (red and blue) components of the basis vector. (TIF) [file pcbi.1008973.s005.tif]

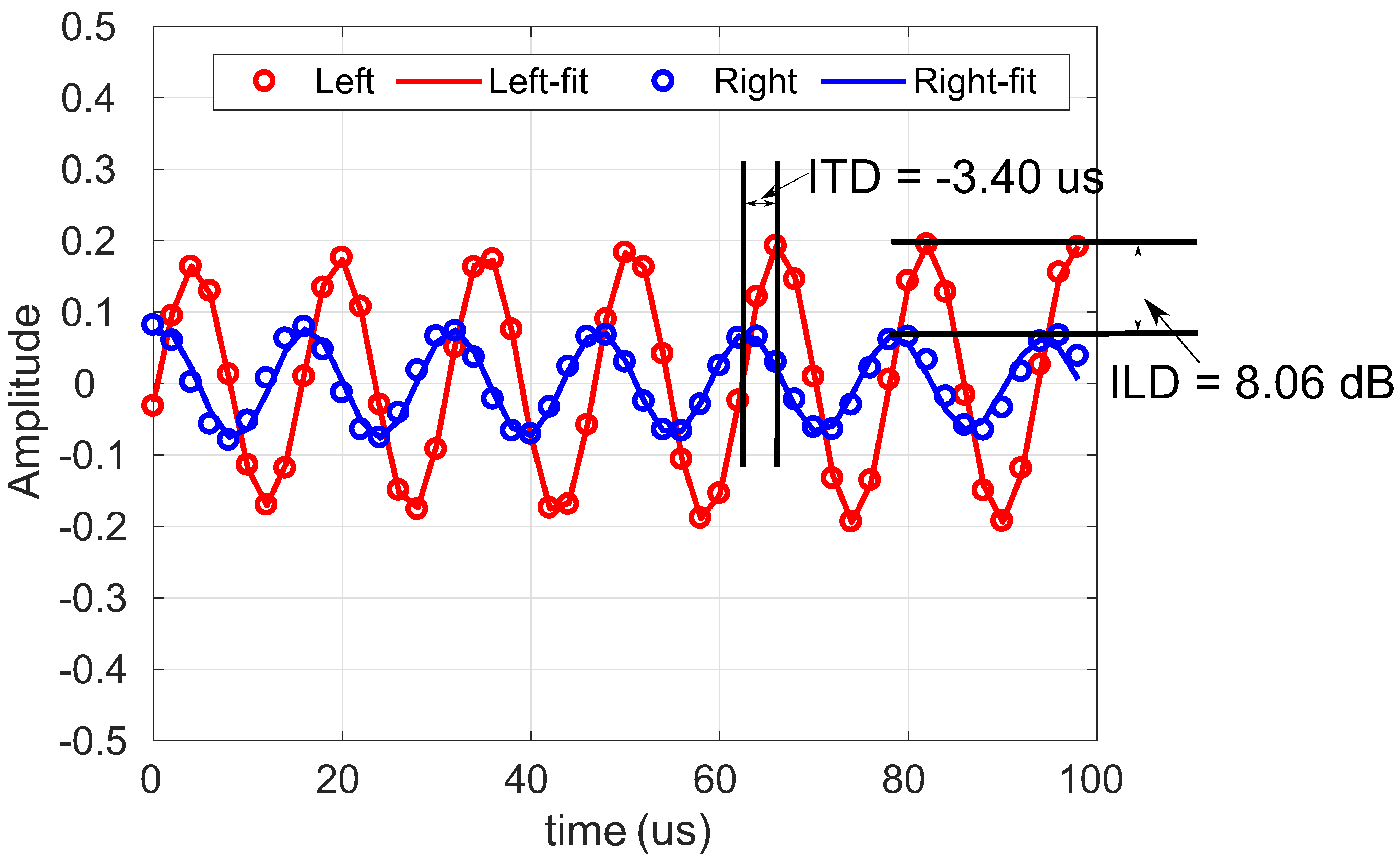

Supplement: S5 Fig — The figure shows the left and right(red and blue dot) components of the basis vectors. The corresponding least squares fit of the function g(t) to the basis vectors here shown by red and blue lines. For the given example, Interaural Level Difference (ILD) = 8.06 dB, Interaural Time Difference (ITD) = -3.40 us, center frequency(fc) = 64.2 kHz and sweep rate(m) = -19.94 kHz/ms. (TIF) [file pcbi.1008973.s006.tif]

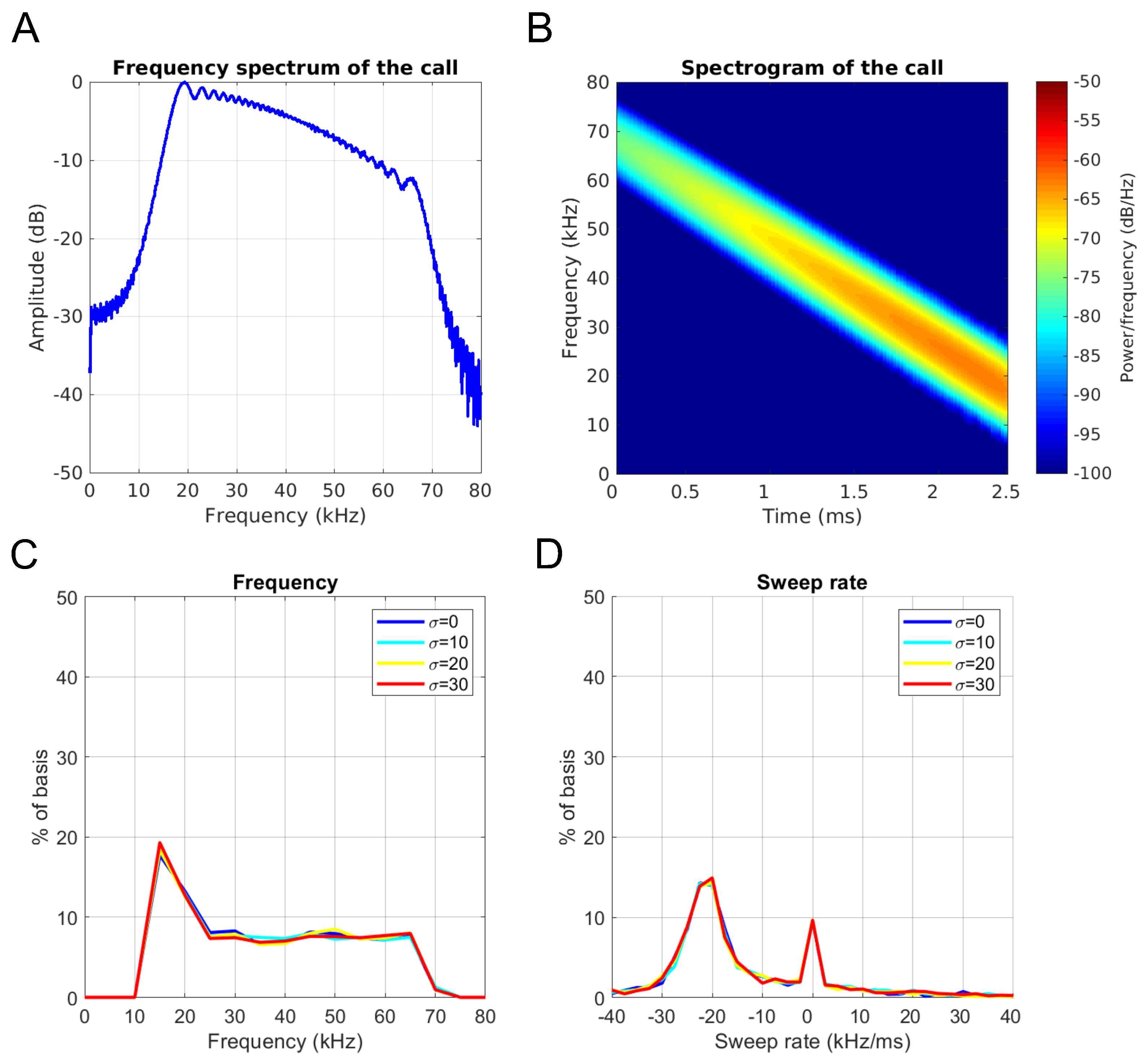

Supplement: S6 Fig — (A) Frequency spectrum of the call. The frequency range of the call is conceptually similar to that of the big brown bat. (B). The spectrogram of a 2.5 ms long call. (B). The distribution of the frequency of the binaural basis vectors. (C). The distribution of the sweep rate of the binaural basis vectors. (TIF) [file pcbi.1008973.s007.tif]

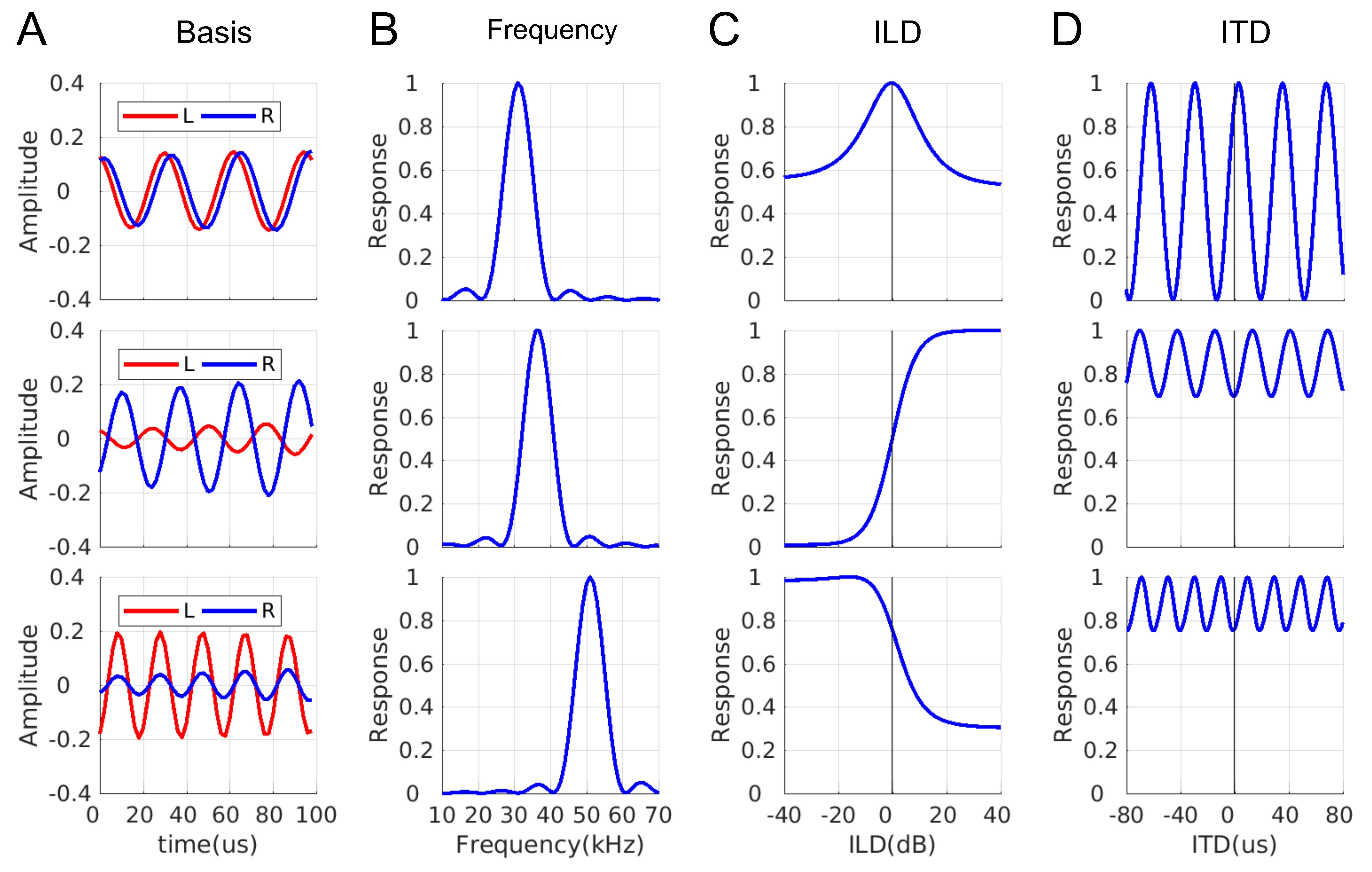

Supplement: S7 Fig — (A) Binaural basis vectors. The left and right (red and blue) components of the basis vector are shown together. (B). The frequency tuning of each binaural basis vector. The basis vectors prefer frequencies with higher responses. (C). The pure tone response to interaural intensity differences. In the figure there are peaked and monotonic response functions (D). The pure tone response to interaural time differences. The shown responses are cyclic. (TIF) [file pcbi.1008973.s008.tif]

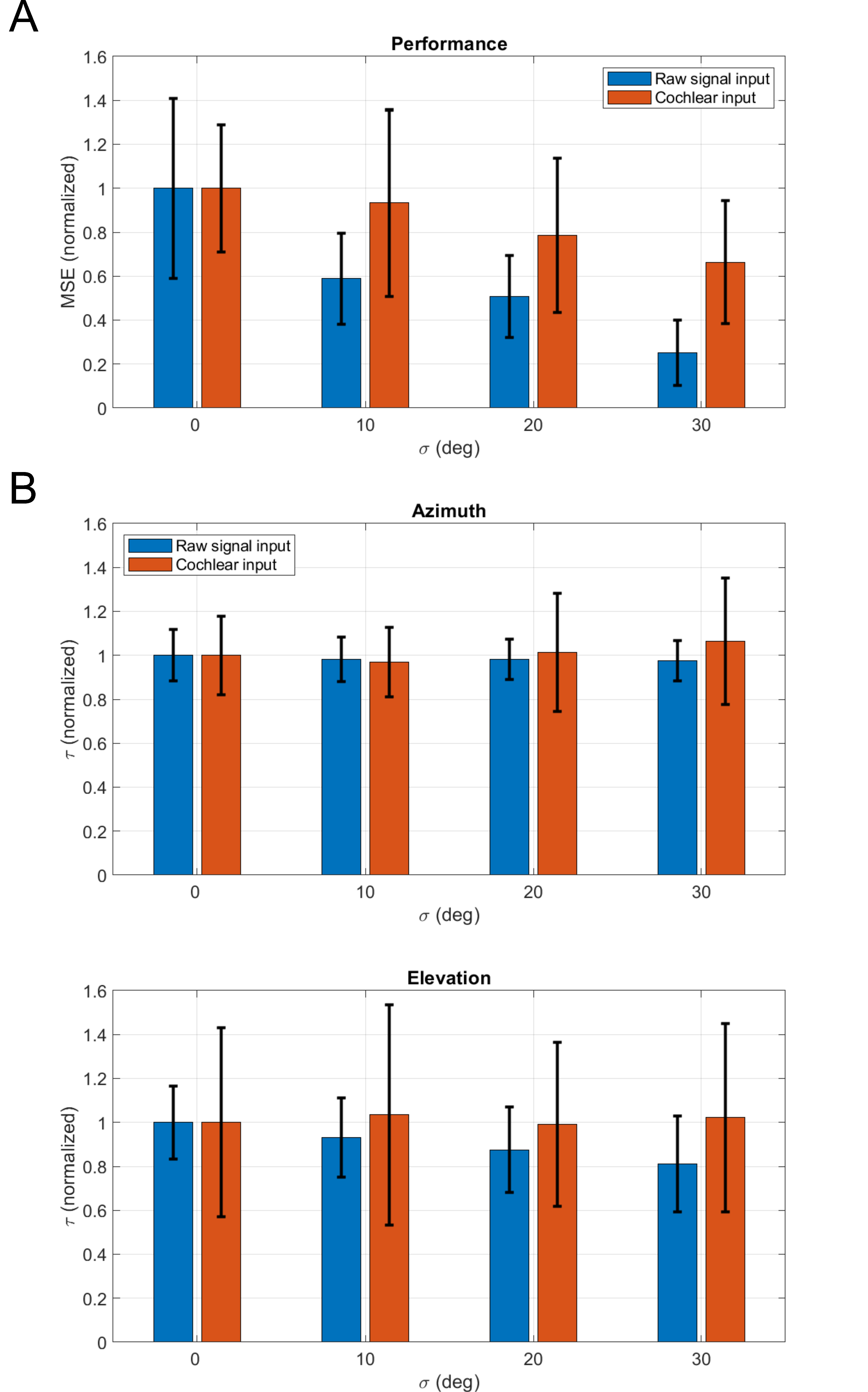

Supplement: S8 Fig — (A) Normalized mean squared error at steady state. (B). Normalized time constant in azimuth and elevation direction. The performance is compared between the two different inputs, raw auditory signals and cochlear responses (blue and red). The error bars show the standard deviation. (TIF) [file pcbi.1008973.s009.tif]
