# Supplementary material for: Active head rolls enhance sonar-based auditory localization performance
Source: PLoS Comput Biol. 2021 May 10;17(5):e1008973. doi: 10.1371/journal.pcbi.1008973 (PMC8136848; doi:10.1371/journal.pcbi.1008973)
Supplement: Attachment — Submitted filename: Response_to_reviewers.docx [file pcbi.1008973.s010.docx]

# Dear Editor and Reviewers,

# We would like to thank the editor and the reviewers for the extensive comments and suggestions. In our responses to each point made by the reviewers, reviewer comments are shown in black. Responses and the modifications are shown in blue and red color fonts respectively.

We thank you for your time and consideration.

Yours sincerely,

Lakshitha P. Wijesinghe,

Graduate Student,

Department of Electronic and Computer Engineering,

Hong Kong University of Science and Technology,

Hong Kong.

# **Reviewer #1:**

This paper presents an improved model for sound source localization in bats. The problem is well motivated, the method appears technically sound, and technical material is very clearly presented. The authors are talented writers and the prose is easy to follow.

I would encourage the authors to provide a bit more narrative structure to the paper. In the introduction, be sure to clearly articulate the goals of the paper and what the reader should expect to gain from reading it, then outline how the paper will lead them there.

**Modification:**

We added a paragraph to the introduction of the manuscript at lines 18-25. We also present an outline for the paper to help the reader navigate through the article at lines 87-93.

Within the body, explain how each component of the model fits into the whole. Because the mathematical model is quite complex with many components, the authors should create more diagrams and figures illustrating how parts of the model interact.

**Modification:**

We updated Fig 2 to better illustrate our architecture. Fig 2A is updated to show the modules that correspond to the subsections under system architecture. Fig 2B is added as a better illustration of the input, output of the GASSOM. Refer to Fig 2.

The discussion section seems to repeat background information more than it discusses the results of the experiments. The discussion should highlight the insights gained from the experiments. Discuss how the proposed model performs compared to prior models.

**Modification:**

We revised the discussion to focus on the insights resulting from our work in the context of past studies. For example, previous research findings have suggested that active head movements may contribute to auditory localization, which we directly explored in this paper. Prior work that highlighted the role of head movements in localization is discussed at lines 448-453. Prior localization models that use the known target direction for learning are described at lines 461-463. Finally, we compared our model with another model that uses cochlear pre-processing of the input in lines 403-434.

The authors should also discuss the specific implications of the work for engineers designing localization systems. They allude to that as a subject for future work, but I see no reason not to address it in this paper.

**Modification:**

We added a paragraph to the discussion at lines 604-611 to include specific implications of our model for engineering designs. We also refer to specific engineering applications in the literature where our model can be applied to.

# **Reviewer #2:**

I agree with the authors that the AEC framework as applied here is very interesting and shows how the mapping of acoustic cues onto task-relevant behavior could be implemented through an unsupervised learning mechanism. However, I’m not sure I concur with the author’s claim: to have gained key insights to sound localization cues used by echolocating bats employing active head movements. I believe that for this claim to be really convincing two limitations to this study should be remedied first: replace the biologically implausible input signals by more plausible ones and present evidence that the sequential localization behavior as predicted by the model is actually observed in real bats.

Regarding the input signals:

In the manuscript microphone signals are used as input to the GASSOM units, but in the real bat, such information is not available. One window-length has approx a duration of 0.1msec (fine), 0.2msec (coarse), sample rate 250k samples/sec. However, the output of the cochlea does not vary at that high rate (LP-filter with cutoff approx 1-3kHz), so the bat’s brain could not analyse the echo signal at this temporal scale. To be biologically plausible the input should be a cochlear-like representation.
**Reply:**

We implemented the cochlear-based feature extractor using the code we obtained from you. In the implementation, we used 81 bandpass filters from 20kHz to 100kHz. The microphone signal is first filtered using the bandpass filters. The filtered output is then sent through a half-wave rectifier and a low pass filter with a cutoff of 1kHz. The output of the low pass filter is named the cochlear representation throughout this description.

The cochlear representation contains spectrotemporal information extracted from the auditory data. We extracted time overlapping spectrotemporal windows from this representation. The time duration of the window is 100us and the stride is selected to be 10us. The window duration and the stride are similar to the proposed approach in our paper. Each window is then downsampled by a factor of 10 to generate an 810-dimensional feature vector for a given ear.

The spectrotemporal data is used as the input to the GASSOMs. In this implementation, we used three GASSOMs for binaural, left, and right monaural data. The responses from the GASSSOMs are pooled over time using average pooling to map them to the head control commands. Similar to our approach, both the representation and the action network are learned to jointly minimize the reconstruction error in encoding.

Our experiments revealed that our main finding was preserved. The MSE decreases with the introduction of active head rolls in both models (i.e. with and without cochlear processing). One difference between the two models is that localization speed improves with the head rolls in our model, but not with cochlear processing. The main difference is that the basis vectors with the cochlear representation only respond to ILD, not to ITD.

**Modification:**

We added a new section “Comparison with a model with cochlear type preprocessing” to the paper to describe the cochlear-based representation in detail. Also, we provide a comparison between the models. Lines 403-434.

Line 154: It would be interesting to see what features these cells would learn with more biologically plausible cochlear input instead of microphone signals.

**Reply:**

We analyzed the representation learned with the cochlear-based pre-processing. The basis vectors in this representation combine information from multiple frequencies received from the channels. In terms of interaural differences, we identified that these cells only respond to interaural level differences. The response did not vary with interaural time differences.

**Modification:**

Lines 403-434.

Line 277: The basis vectors are stated to correspond with auditory receptive fields in the mammalian auditory pathway. Please provide a reference for bats to support this statement and argue why, despite the implausible input signal, this would still be the case.

**Reply:**

We decided to add a new subsection to the paper titled “Comparison with biological sound localization mechanisms”. The purpose of this section is to compare the properties of the learned representation with the properties of the cells in the auditory pathway.

In particular, we compared the ILD distribution with the ILD distributions provided in the literature. Also, we compared the types of responses we observe in our model and the cells in the auditory pathway given the same binaural stimuli.

**Modification:**

The new section “Comparison with biological sound localization mechanisms” at the lines 355-380.

Regarding the sequential nature of the localization process:

Line 130: The system updates its head orientation for 20 iterations (=20 call-echo pairs). How was this value chosen? Does it have biological relevance? In reality, the target direction relative to the bat will change over calls as either the prey or the bat and usually both will move in between calls. How would that affect the proposed localization scheme?

**Reply:**

In the bat experiment, the bat localizes targets that approach the bat along the range axis. The distance between the bat and the target decreases over time (for the simple target motion condition). We selected a 1-2.5 m distance as the range where the bat localizes the target. We counted the number of calls that the bat emitted during this period and the Pulse Interval (PI) between successive calls in this range. The average pulse interval is 82.3 ms and the number of calls is 16$\pm$7. The choice of 20 call-echo pairs in our experiment approximates the number of samples used by the bat in the behavioral tracking experiment.

In the experiments we propose here, the target is assumed to be stationary during the 20 call-echo pair. Our model should be able to track dynamic auditory targets to align the head directly toward the target irrespective of the changing distance between the bat and the target. The reason is that we normalize the energy of the received echo.

In future research, we intend to extend our model to track a moving auditory target. We also plan to model moving target interception by combining the tracking with flight dynamics. This idea is presented as the future work of this model.

**Modification:**

We added a description of the parameter selection to lines 141-148.

Line 268: The MSE is evaluated after 20 iterations. It is not clear to me that this is the most sensible performance characteristic to evaluate. As the bat’s localization seems more a compromise between speed and accuracy, it seems more logical to evaluate how many vocalizations are required before the target is localized with a behaviorally relevant accuracy (3° for example). As mentioned above, the bat has to localize in a dynamic context where relative prey position is possibly changing fast (in particular at close range) so localization speed (given a certain accuracy) seems to me the foremost performance criterion. It is also not clear to me what head-roll speeds are resulting from the proposed mechanism? What measurement rate (call-echo iterations per second) is assumed?

**Reply:**

In the results section, we demonstrate two different types of improvement in localization behavior. First, we demonstrate that the localization speed improves with the introduction of head rolls. Second, we illustrate that the steady-state error (recorded at the end of 20 interactions) decreases with active head rolls. To demonstrate the localization speed, we estimate the time constant from the head trajectories. Although the time constant does not directly indicate the time required to achieve a fixed accuracy, we feel it is a better measure. The time to reach a fixed accuracy depends upon the initial error, whereas the time constant is independent of the initial error. Rather, it is a widely used measure reflecting the time it takes for the error to reduce by 63%. From our results, the time constant decreases with the standard deviation of the head rolls when localizing targets in elevation.

**Modification:**

We added the implication of the time constant to the behavior. Lines 303-308.

Specific comments:

Line 28-29: Did the bat’s ears move with respect to its head in the ‘bat experiment’-measurements used here? If so, the extracted head orientation (Fig. 1) seems problematic as it would be affected by non-rigid movement of pinnae with respect to the head? Indeed, from fig S1 and the explanation on line 92, it can be concluded that movement of the pinna tips will affect the estimated head orientation.

**Reply:**

Yes, In the bat experiment, the bats adjusted inter-pinna separation with changes in target distance. They either raise the tips of the pinnae to decrease the inter-pinna separation (at a longer target distance) or lower the tips of the pinnae (at a shorter target distance) to increase the inter-pinna separation. If these movements are symmetric and in the same plane as defined by the initial pinna tips and head marker, the orientation estimate will not be affected. Otherwise, we agree with the reviewer that there will be small errors in the estimated head orientation. However, these errors do not change our primary conclusion, namely that head rolls improve localization accuracy in the vertical plane. We do not use experimentally measured head trajectories in our experiments, but rather use them to estimate the parameters of the statistical model used to generate random head movements. While the exact parameter values would change, our experimental results indicate that the reduction in MSE occurs over a broad range of parameters.

**Modification:**

We added a description of the motion of the pinna to the caption of the S1 Fig. at lines 774-780.

Line 60: Why use azimuth, elevation, and roll angles to characterize the head orientation (re. body/world), as the literature mostly uses yaw, pitch, and roll?

**Modification:**

We replaced the azimuth, elevation with yaw and pitch at lines 63-64.

Line 98: The Fick gimbal system: Please define the world (=body?) coordinate system you use. Fig. S2 is somewhat ambiguous. Is the angle alpha defined by the intersection of the cone of confusion around the ear-ear axis with the horizontal plane (Eq. 1)? But if the head is rolled, what you refer to as the horizontal plane will then rotate around what you call the head direction and will no longer be the true horizontal plane, or not?

**Reply:**

We updated Fig 2 to illustrate the body (B) and head (H) centered coordinate axis.

Regarding angle alpha- Yes, the alpha angle is defined by the intersection of the cone of confusion with the horizontal plane.

Regarding head rolls- As the head rolls, the horizontal plane will rotate around the head direction axis as it is defined in the head-centered coordinates.

**Modification:**

We modified Fig 2 to include the body (B) and head (H) centered coordinates. The axis in S2 Fig. is also updated to indicate the head-centered coordinates.

We included a definition of alpha in the caption description of S2 Fig. Also, the rotation of the horizontal plane around the head direction is described in the caption of S2 Fig.

The coordinate system used most often In acoustics is: the ear-ear axis (y-axis), the up direction (z-axis) and the forward direction (x-axis), with the last two in the mid-sagittal plane and defined fixed to the head. It seems this is the coordinate system the HRTF was measured in or am I mistaken?

**Reply:**

Yes, we use the same coordinate system, ear-ear axis (Y-axis), the up direction (Z-axis), and the forward direction (X-axis). It is the same coordinates used in HRTF measurements.

**Modification:**

We updated Fig 2 and S2 Fig. to indicate the XYZ axis. We also updated the captions of the figures to describe the axis of the coordinate system.

Line 142: Please note that you left out the spatio-spectral properties of emission despite sonar being an active sensor system. Hence, as the emission system also has a frequency dependent directivity it should be combined with the HRTF to simulate biologically plausible echo signals. Fundamentally, this wouldn’t change anything but please comment on how you expect this would affect your set of basis functions?

Reply:

Yes, in our experiment, we did not consider the directionality of the call emission. The reviewer is correct that this would change the statistics of the received signals, and thus the precise shape of the basis functions. If the call emission direction is held constant in the forward direction, then taking the spatio-spectral properties of emission into account would result in a systematic change in the received acoustic signals that could be modelled by systematic changes in the HRTF. We expect that changes in the basis would be small, since once the localization behavior is learned, the target location will be biased to lie mostly directly in front of the bat, which reduces the variability of the received signals. If the call emission direction varies, then this would introduce some variability in signals received from a given target location in head centered coordinates. Even in this case, we would expect that changes in the basis function to be small, for the same reason as above. Once localization is learned, the target lies mostly in front of the bat, so we would expect most emissions to be directed in the forward direction. The bigger effect would be on the behavior of the model, which does not take into account changes in the emission direction. If the controller had access to the emission direction (as we would expect in the real bat), it could potentially compensate for these changes. If it did not, then changes in emission direction can be considered as a noise source, which we expect would degrade the speed of localization.

**Modification:**

Lines 533-550.

The simulated echo signals seem to contain only a prey echo and no clutter echoes? Also, it seems that a prey echo is modeled as a point reflector or is there also a reflection filter taken into account? To judge the biological plausibility of your proposed mechanism it would be interesting to know your thoughts on how these more realistic conditions would affect your learning mechanism and how it could deal with their possibly adverse effects?

Reply:

In our simulations, we only have a prey echo and the prey is modeled as a point reflector. Echoes received from objects with larger extent and at different depths can be modeled as a superposition of multiple delayed echoes. Our model covers the localization phase, where we assume the bat has selected the target and is approaching it. Thus, we expect the target distance to be decreasing so that it is generally the closest object and the emission direction is in the direction of the object which is towards the front of the bat. Here, the spatio-spectral properties mentioned above would be a benefit, as the intensity of the emission drops of with eccentricity, reducing the magnitude of echoes from other objects. In this setting, the magnitude of echoes from other objects would be reduced. Thus, we expect that the learning mechanism would still be effective. Localization accuracy would be similar, but we expect that localization speed would be degraded, as the additional objects would introduce noise, albeit with smaller magnitude than the target echo. An interesting extension of this work suggested by the reviewer’s comment would be to incorporate a learned attention mechanism that could to further improve performance by focusing on aspects of the signal that are most relevant to the target, e.g. along the lines of previous work we have done in the visual domain.

**Modification:**

Lines 551-566.

Line 184: f(t) is obtained by pooling the GASSOM outputs. Please state what kind of pooling you did: maximum pooling or something else?

**Modification:**

We state that we use average pooling in lines 216-218.

Line 216: What is the reasoning behind using Listings’s law and eq. (8). It is not clear to me why the mean head roll angle should be a function of the head direction (re. body) angles?

**Reply:**

There are two reasons behind using Listing’s law. First, we desire to have the same mean head roll angle for a given head direction during behavior. The 3D head orientation for a given head direction in azimuth and elevation depends on the actions taken to reach the head direction. To have the same mean 3D head orientation, we define the head roll around the roll angle given by Listing's law. The random head rolls are added to the head roll angle from Listing’s law to simulate active head rolls. Second, the roll angle defined by Listing’s law also is the 3D head orientation that optimizes motor efficiency. Although Listing’s law is commonly used to describe eye movements, there is evidence that Listing’s law also applies to gaze-directing head movements.

**Modification:**

We include the reasoning for applying a mean head roll angle to lines 258-265.

Line 238: It is not entirely clear whether active head rolling is limited to the training phase or whether it also occurs during testing?

**Reply:**

We only apply the active head rolls in the training phase.

Line 290: Fig. 5B shows the distribution of IID and ILD tuning of binaural basis vectors. Does this correspond with what is known about the distribution of such cells in the bat brain? If (not) so, this could be indirect evidence (against) in favor of the biological plausibility of the proposed approach.

Also, one wonders to what extent the tuning of the majority of binaural basis vectors to zero IID and ILD values is due to the reinforcement learning paradigm used. As the system is trained to home in on a perceived target it will collect very few echoes with large IID and ITD values and many with small IID and ITD values (as every track takes 20 iterations, most of which will point to a direction close to the target). I would be interested in the author’s thoughts about this point?

**Reply:**

ILD and ITD distributions - In our analysis, we compared the distribution of ILD, ITD for our binaural basis to the distribution of cells in the auditory pathway. We updated the paper to compare our ILD distribution and the distribution of ILD reported for bat IC cells. We are including quantified similarities between the ILD distributions in the paper. ITD responses recorded from the bat auditory pathway show certain differences with the ITD responses we report in this paper. We discuss these differences in detail in the discussion.

The shape of the distribution due to the learning paradigm. We agree with the reviewer that the statistics of the input are shaped by the reinforcement learning paradigm (and more generally by behavior). Indeed, modelling the interplay between perception and behavior is one of our primary motivations in developing the AEC paradigm. Due to the developed behavior, the ITD and ILD tunings of the basis functions are expected to have values closer to zero. We mentioned the advantages of this bias in our responses above to the reviewer’s comments regarding the directionality of call emission and the superposition of echoes from multiple spatially extended targets.

**Modification:**

We added a comparison of the distributions to the results. We also describe the reason for the shape of the distribution using our learning paradigm. Lines 340-349.

The similarities and differences in comparison to biology are also discussed in detail in lines 567-580.

Line 332 and 338: ‘The recent discovery that bats exhibit head waggles as they track sonar targets …’. I’m not sure the claim in line 338 is justified. At least in the context of obstacle avoidance this insight has been around for a while. See ‘Mogdans J, Ostwald J, Schnitzler H. The role of pinna movement for the localization of vertical and horizontal wire obstacles in the greater horseshoe bat, Rhinolopus ferrumequinum. The Journal of the Acoustical Society of America. 1988;84(5):1676–1679. doi:10.1121/1.397183.’ and ‘V. A. Walker, H. Peremans, and J. C. T. Hallam, One tone, two ears, three dimensions: A robotic investigation of pinnae movements used by rhinolophid and hipposiderid bats, The Journal of the Acoustical Society of America. 104 (1), July 1998: 569-579’ for a computational analysis of what cues such behavior gives rise to. In both these papers the primary focus is on pinnae movements but head waggling by bats is proposed in both as an alternative way of generating very similar cues.

**Reply:**

Yes, we agree with the fact that previous papers have suggested that head movements contribute to localization behavior. Our model is primarily based on the behavioral experiments that have revealed that bats use head waggles when localizing an approaching target (Wohlgemuth et al., 2016). Also, in obstacle avoidance, CF bats increased head movements when the pinnae were immobilized (Mogdans et al., 1988). Head tilting in the lateral plane has also been proposed as a mechanism to generate elevation-dependent cues (Walker et al.,1998). We think our model provides more comprehensive evidence using the computational model.

**Modification:**

We added the references suggested by the reviewer into our discussion to acknowledge the previous behavioral experiments and ideas that propose a similar hypothesis. Lines 448-453.

Typo’s:

Line 70: also

**Modification:**

We deleted “also.” The updated sentence can be found on lines 73-75.

Line 103: computer

**Modification:**

We changed “computer” to “computed” on lines 115-116.

Ref 26: something went wrong with the author list. Also, I believe a more complete and more easily available reference to the ideas discussed there would be: ‘B. Fontaine, H. Peremans, Bat echolocation processing using first-spike latency coding, Neural Networks 22 (2009) 1372–1382’

**Modification:**

We added a citation to the journal article “Bat echolocation processing using first-spike latency coding” along with the citation to the conference paper. The updated reference can be found on line 464.

Fig. S3: in the legend: ‘Fiting’

**Modification:**

Legend of the S3 Fig. is updated to change “Fiting” to “Fitting”.

# **Reviewer #3:**

Software:

1. I downloaded the zipped programs from https://github.com/HKUST-NISL/Echolocation-AEC, but there is a missing file “HRTF_2.mat” that prevents the program from running.

**Reply:**

We uploaded all the HRTF data files to the HRTF folder. Please find the updated data at the link <https://github.com/HKUST-NISL/Echolocation-AEC>

2. azimuth, elevation, and roll angles on line 60 are confusing because there are pitch, yaw, and roll in aircraft axes. Do the azimuth, elevation, and roll correspond to yaw, pitch, and roll?

**Modification:**

We replaced the azimuth, elevation with yaw and pitch at lines 63-64.

3. Line 70: there are two “also”.

**Modification:**

The repeated on “also” at lines 73-75.

4. Reconstruction error is an important concept throughout the paper, but is too abstract to understand with equation (3). Can the authors provide more explanation when it first shows up in the manuscript or at least provide a citation that describes it? In the citation for GASSOM model – citation #19, there is no reconstruction error.

**Reply:**

We added a detailed but simple explanation of the reconstruction error to the manuscript. We expect the reader could grasp the main concept through this description. We explain that the bat receives echoes from different directions. The perceptual representation encodes the auditory signals received. There is an error associated with the encoding, we define it as the reconstruction error. Before the encoding process, we divide the received auditory signal into windows. Eq. 3 describes the reconstruction error for the auditory stimuli for a given scale s, ear e, and window i.

**Modification:**

We added a simplified description to lines 200-209.

5. a. The information for training the action network is not enough. Line 192: The hidden layer activations are then mapped to two scalar outputs corresponding to azimuth and elevation. Are the two scalar outputs the motor command? It looks like they are in Fig. 2.

**Reply:**

We expanded the description for action network training to the following.

The action network maps the feature vector f($t$) from the representation stage to motor commands $\Delta\theta$(*t*) and $\Delta\phi(t)$. These motor commands update the bat's head direction in azimuth and elevation. We use a reinforcement learning algorithm to learn a head control policy. In our algorithm, we consider f($t$) to be the state and the motor commands $\Delta\theta$($t$) and $\Delta\phi(t)$ to be the actions.

The feature vector f($t$) is fed into an artificial neural network with a hidden layer comprising 500 tanh nonlinear activation units. The hidden layer activations are then mapped to two scalar outputs corresponding to azimuth and elevation head motor commands.

**Modification:**

We provide the expanded description at lines 220-225.

b. Line 196: During training, the motor commands are sampled from a Gaussian distribution with mean equal to the output of the network. My understanding is in training, you teach the network with input, and true output. How are the “true” values of “the two scalar outputs” determined in training?

**Reply:**

We use a reinforcement learning algorithm to learn the head control policy. The true motor commands (labels) are not required for training the network. We use random actions to explore the action space.

**Modification:**

We added a sentence to describe the way we train the model without the requirement for the actual labels of the motor outputs at line 226.

c. Why use a different greedy policy in testing? What benefits are there?

**Reply:**

During training, a policy with some stochasticity is required to enable to reinforcement learning to explore the policy space. However, this stochasticity degrades performance if an optimal policy has been learned. In reinforcement learning, this is referred to as the “exploration-exploitation’ tradeoff. We do not add randomness in testing because this degrades performance according to the standard deviation of the Gaussian, which is somewhat arbitrary. Our results from testing (where the standard deviation is 0) can be considered as an upper bound on the performance.

**Modification:**

The reason for using a greedy policy in the training phase is described at lines 236-241.

6. Control of head direction in methods is not in Figure 2 System Architecture. Without looking at your code, is it parallel to action network? Where should it be in the architecture?

**Reply:**

The head direction control is the next stage after the action network. The head direction control stage depends on the azimuth, elevation motor commands, and the randomly sampled head roll angle.

**Modification:**

Fig. 2 is updated to include the control of head direction. We hope the reader could get a better association with the subsections in the text and the components of the model.

7. In figure 2, if the five empty boxes denote the other 5 GASSOM units, why not number them GASSOM 1, GASSOM 2, and so on? Or write GASSOM UNITS on the top of the first box, and write numbers in the boxes.

**Modification:**

Fig. 2 is updated to include the numbered GASSOM units. The caption of the Figure is also updated to indicate this enumeration.

8. Even there are insets that zoom in at the center, it is still difficult to figure out how each trajectory converges to the center. Maybe use different color for each target?

**Modification:**

We updated Fig. 3 to indicate target trajectories by different colors. Also, we changed the marker that indicates the point attractor.

9. What is the evidence for the conclusion on line 238 - “the active head rolls enhanced the target localization”? What is it compared to?

**Reply**

In this section, the main idea is to illustrate the performance gain qualitatively. We agree that the topic sentence in this paragraph precedes the evidence we provide in the section. We updated the paragraph such that we describe our data generation approach.

**Modification:**

We removed the topic sentence of the paragraph as given in line 276.

# **Reviewer #4:**

In this manuscript, the authors studied how active head roll movements can aid sound localization in the context of echolocation by the big brown bat. More specifically, they sought to investigate how an autonomous model can learn to align the bat’s head direction to a target of interest. This paper is very well written, addressing clearly articulated questions, with modelling well-laid out and executed, and containing rigorous analysis. The proposed model is well thought out, concordant with previous behavioral work. Overall, the study is of high quality and therefore should be published.

Some very minor comments

- Page 4: 1234? Echoes. What a nice numerical symbology coincidence

**Reply:**

Yes, we considered echoes collected from three bats. The summation happens to be 1234.

**Modification:**

We updated the line 115-116 to indicate that the number of echoes is summed over three bats.

- Page 4: “assume the head assumes” reads weird

**Modification:**

We updated the line 132-133.

- Page 5: When first mentioned, HRTF is not defined

**Modification:**

We updated line 138-140 so that HRTF is expanded at the first use of the term.

- According to SI style conventions, there should be a space between a numerical value and unit symbol.

**Modification:**

We added a space between the numerical value and the unit symbol at all the occurrences.

- Active movements may also perturb sensory representations (for vision, retinal movement blurs the sensory representation; coordinate transformations and spatial updating all require accurate sensory and predictive information). The authors may consider mentioning this briefly.

**Modification:**

We have added a short discussion of this to the discussion at lines 511-517.

- Similarly, there may be energetic and physical constraints to head roll motion, which is not mentioned. May this be a reason why the measured

**Reply:**

We discuss several reasons that the measured head roll standard deviation is lower than the values we experiment in our approach. The reasons are discussed in line 518-529.
